# A new statistical methodology using the sine function: Control chart with an application to survival times data

**DOI:** 10.1371/journal.pone.0285914

**Published:** 2023-08-17

**Authors:** Mustafa Kamal, Gadde Srinivasa Rao, Meshayil M. Alsolmi, Zubair Ahmad, Ramy Aldallal, Md. Mahabubur Rahman

**Affiliations:** 1 Department of Basic Sciences, College of Science and Theoretical Studies, Saudi Electronic University, Dammam, Saudi Arabia; 2 Department of Mathematics and Statistics, University of Dodoma, Dodoma, Tanzania; 3 Department of Mathematics, College of Science and Arts at Khulis, University of Jeddah, Jeddah, Saudi Arabia; 4 Department of Statistics, Quaid-i-Azam University, Islamabad, Pakistan; 5 Department of Accounting, College of Business Administration in Hawtat Bani Tamim, Prince Sattam Abdulaziz University, Al-Kharj, Saudi Arabia; 6 Department of Statistics, Islamic University, Kushtia, Bangladesh; Central Queensland University, AUSTRALIA

## Abstract

Statistical methodologies have a wider range of practical applications in every applied sector including education, reliability, management, hydrology, and healthcare sciences. Among the mentioned sectors, the implementation of statistical models in health sectors is very crucial. In the recent era, researchers have shown a deep interest in using the trigonometric function to develop new statistical methodologies. In this article, we propose a new statistical methodology using the trigonometric function, namely, a new trigonometric sine-*G* family of distribution. A subcase (special member) of the new trigonometric sine-*G* method called a new trigonometric sine-Weibull distribution is studied. The estimators of the new trigonometric sine-Weibull distribution are derived. A simulation study of the new trigonometric sine-Weibull distribution is also provided. The applicability of the new trigonometric sine-Weibull distribution is shown by considering a data set taken from the biomedical sector. Furthermore, we introduce an attribute control chart for the lifetime of an entity that follows the new trigonometric sine-Weibull distribution in terms of the number of failure items before a fixed time period is investigated. The performance of the suggested chart is investigated using the average run length. A comparative study and real example are given for the proposed control chart. Based on our study of the existing literature, we did not find any published work on the development of a control chart using new probability distributions that are developed based on the trigonometric function. This surprising gap is a key and interesting motivation of this research.

## 1 Introduction

Probability distributions have great and significant roles in analyzing the random phenomenon in every sector of life. However, more useful efforts are still needed to look for more flexible probability distributions for data modeling in several areas such as the medicine/biological sector, engineering (modeling reliability phenomena), economics (modeling and predicting import and export), genetics, agronomy; see Rao and Aslam [1], Strzelecki [2], Tung et al. [3], Reynolds et al. [4], and Prataviera [5]. Thus, researchers aim to develop new probability models that are able to provide the best description of the phenomenon under consideration, so we (or new researchers) can have a better and easy understanding of the factors involved.

In order to attempt the probability distributions more flexible, most of the developed methods (or probability distributions) have a higher/large number of parameters. Hence, more computational work is required to obtain the estimates and distributional properties of such distributions; see Souza et al. [6]. Therefore, it is desirable to develop new methodologies or new probability models that have a small number of additional parameters and provide greater distributional flexibility in data modeling with a large degree of best fitting; see Thach and Bris [7], Starling et al. [8], and Giles [9].

In order to reach the above aim, a number of researchers have introduced new methodologies for developing new probability distributions. However, few researchers have focused on using trigonometric functions to develop new distributions. In this regard, Kumar et al. [10] incorporated a trigonometric function to develop a new method for generating new probability models. For this purpose, they used a sine function and called the proposed method as SS transformation. The cumulative distribution function (CDF) of their proposed method is given by

$$\begin{array}{c} F(t;\;\kappa )=\text{sin}\;[\frac{\pi }{2}G(t;\;\kappa )],\;\;\;\;\;\;t\in R, \end{array}$$ (1)

with probability density function (PDF) given by

$$\begin{array}{c} f(t;\;\kappa )=\frac{\pi }{2}g(t;\;\kappa )\;\text{cos}\;[\frac{\pi }{2}G(t;\;\kappa )],\;\;\;\;\;\;t\in R, \end{array}$$ (2)

where $g(t;\;\kappa )=\frac{d}{dt}G(t;\;\kappa )$ and ***κ*** is a parameter vector associated with *G*(*t*; ***κ***).

The trigonometric function, defined in Eq (1), was further extended by (*i*) Mahmood et al. [11] by developing a new sine-*G* family, (*ii*) Al-Babtain et al. [12] proposed the Sine Topp-Leone-*G* family, (*iii*) Jamal et al. [13] by introducing the sine extended odd Fréchet-*G* family, and (*iv*) Jamal et al. [14] by studying the transformed Sin-*G* family.

As per our search and knowledge, very limited work has been done using the trigonometric function. However, to the best of our knowledge, there is no published work on the development of a control chart using new probability distributions that are developed based on the sine function. In this paper, we introduce a new method for generating new families of distributions using the sine function. We call the proposed method as a new trigonometric sine-*G* (NTS-*G*) family of distributions. Based on the sine NTS-*G* approach, we also develop a new control chart and show its application practically. This is one of the key motivations of this work.

**Definition**: Suppose *T* has the NTS-*G* family, if its CDF *F*(*t*; λ, ***κ***), is given by

$$\begin{array}{c} F(t;\lambda ,\kappa )=1-\frac{\lambda (1-\text{sin}\;[\frac{\pi }{2}G(t;\;\kappa )])}{\lambda -\text{sin}\;[\frac{\pi }{2}G(t;\;\kappa )]},\;\;\;\;\;\;t\in R,\lambda >1. \end{array}$$ (3)

Corresponding to *F*(*t*; λ, ***κ***) in Eq (3), the PDF *f*(*t*; λ, ***κ***) of the NTS-*G* family is given by

$$\begin{array}{c} f(t;\lambda ,\kappa )=\frac{\pi \lambda (\lambda -1)g(t;\;\kappa )\;\text{cos}\;[\frac{\pi }{2}G(t;\;\kappa )]}{2(\lambda -\text{sin}\;[\frac{\pi }{2}G(t;\;\kappa )]{)}^{2}},\;\;\;\;\;\;t\in R,\lambda >1. \end{array}$$ (4)

Furthermore, the expressions for the survival function (SF) *S*(*t*; λ, ***κ***), hazard function (HF) *h*(*t*; λ, ***κ***), and cumulative HF (CHF) *H*(*t*; λ, ***κ***) of the NTS-*G* family are, respectively, given by

$$\begin{array}{c} S(t;\lambda ,\kappa )=\frac{\lambda (1-\text{sin}\;[\frac{\pi }{2}G(t;\;\kappa )])}{\lambda -\text{sin}\;[\frac{\pi }{2}G(t;\;\kappa )]},\;\;\;\;\;\;t\in R,\lambda >1, \end{array}$$ (5)

$$h(t;\lambda ,\kappa )=\frac{\pi (\lambda -1)g(t;\;\kappa )\;\text{cos}\;[\frac{\pi }{2}G(t;\;\kappa )]}{2(1-\text{sin}\;[\frac{\pi }{2}G(t;\;\kappa )])(\lambda -\text{sin}\;[\frac{\pi }{2}G(t;\;\kappa )])},\;\;\;\;\;\;t\in R,\lambda >1,$$

and

$$H(t;\lambda ,\kappa )=-\text{log}\;(\frac{\lambda (1-\text{sin}\;[\frac{\pi }{2}G(t;\;\kappa )])}{\lambda -\text{sin}\;[\frac{\pi }{2}G(t;\;\kappa )]}),\;\;\;\;\;\;t\in R,\lambda >1.$$

The very next section offers the expressions of basic functions of the new trigonometric sine-Weibull (NTS-Weibull) distribution. Furthermore, the plots for the density of the NTS-Weibull distribution are also presented. The estimation of the parameters and different simulation studies are provided in Section 3. Section 4 is devoted to illustrate the NTS-Weibull distribution using a medical data set. The development of the control chart along with numerical studies are presented in Section 5. Finally, in Section 6, the article is concluded.

## 2 A NTS-Weibull model

Here, we calculate the basic functions of the NTS-*G* family of distributions. Let *T* has the NTS-Weibull distribution with CDF *F*(*t*; λ, ***κ***), if it is given by

$$\begin{array}{c} F(t;\lambda ,\kappa )=1-\frac{\lambda (1-\text{sin}\;[\frac{\pi }{2}(1-e^{-\alpha t^{\delta }})])}{\lambda -\text{sin}\;[\frac{\pi }{2}(1-e^{-\alpha t^{\delta }})]},\;\;\;\;\;\;t\geq 0. \end{array}$$ (6)

Corresponding to *F*(*t*; λ, ***κ***), the PDF *f*(*t*; λ, ***κ***) of the the NTS-Weibull distribution is given by

$$\begin{array}{c} f(t;\lambda ,\kappa )=\frac{\pi \lambda (\lambda -1)\alpha \delta t^{\delta -1}e^{-\alpha t^{\delta }}\;\text{cos}\;[\frac{\pi }{2}(1-e^{-\alpha t^{\delta }})]}{2(\lambda -\text{sin}\;[\frac{\pi }{2}(1-e^{-\alpha t^{\delta }})]{)}^{2}},\;\;\;\;\;\;t>0. \end{array}$$ (7)

**Fig 1:**
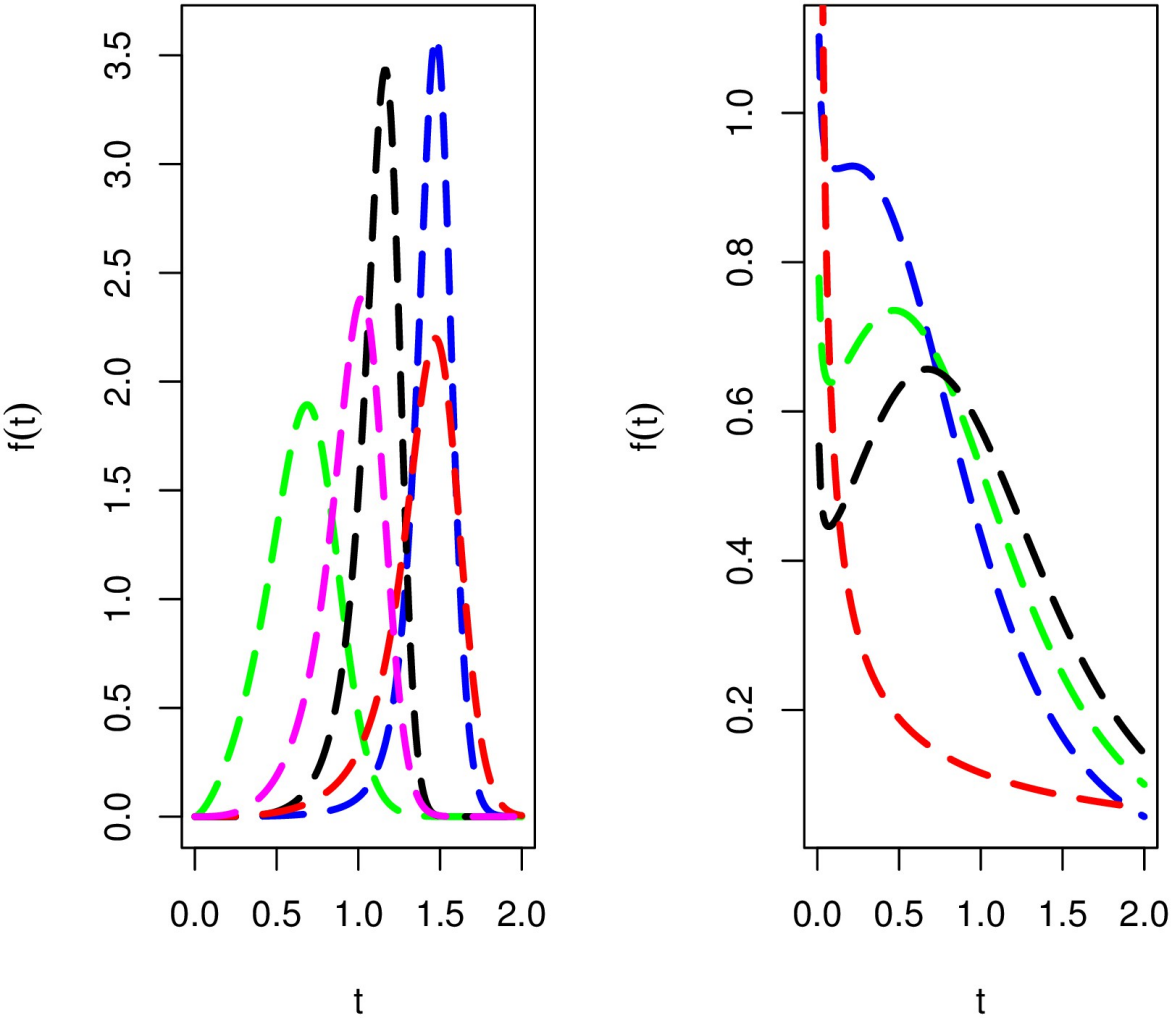
Visual behaviours of *f*(*t*; λ, *κ*) of the NTS-Weibull model.

Fig 1 offers different visual behaviors of *f*(*t*; λ, ***κ***) of the NTS-Weibull model. The plots of *f*(*t*; λ, ***κ***) of the proposed model are obtained for (*i*) *δ* = 4.2, *α* = 0.5, λ = 1.01 (blue curve line), (*ii*) *δ* = 2.5, *α* = 2.2, λ = 1.5 (green line), (*iii*) *δ* = 6.1, *α* = 0.5, λ = 1.2 (black line), (*iv*) *δ* = 4.2, *α* = 0.3, λ = 1.1 (yellow curve line), (*iiv*) *δ* = 4.1, *α* = 1.0, λ = 1.3 (magenta curve line), (*iiiv*) *δ* = 0.85, *α* = 1.4, λ = 1.4 (gold curve line), (*ix*) *δ* = 0.8, *α* = 1.4, λ = 1.2 (red line), (*x*) *δ* = 0.75, *α* = 1.5, λ = 1.1 (brown curve line), and (*xi*) *δ* = 0.2, *α* = 1.1, λ = 1.1 (grey curve line).

Fig 1 shows that *f*(*t*; λ, ***κ***) of the NTS-Weibull distribution has different shapes including (*i*) right-skewed (green curve line), (*ii*) symmetrical (magenta curve line), (*iii*) left-skewed (black, blue, and yellow curve lines), (*iv*) decreasing (grey curve line), and (*v*) decreasing-increasing-decreasing (gold, red, and brown curve lines).

Furthermore, the expressions for the SF *S*(*t*; λ, ***κ***), HF *h*(*t*; λ, ***κ***), and CHF *H*(*t*; λ, ***κ***) of the NTS-Weibull distribution are, respectively, given by

$$S(t;\lambda ,\kappa )=\frac{\lambda (1-\text{sin}\;[\frac{\pi }{2}(1-e^{-\alpha t^{\delta }})])}{\lambda -\text{sin}\;[\frac{\pi }{2}(1-e^{-\alpha t^{\delta }})]},$$

$$h(t;\lambda ,\kappa )=\frac{\pi (\lambda -1)\;\alpha \delta t^{\delta -1}e^{-\alpha t^{\delta }}\;\text{cos}\;[\frac{\pi }{2}(1-e^{-\alpha t^{\delta }})]}{2(1-\text{sin}\;[\frac{\pi }{2}(1-e^{-\alpha t^{\delta }})])(\lambda -\text{sin}\;[\frac{\pi }{2}(1-e^{-\alpha t^{\delta }})])},$$

and

$$H(t;\lambda ,\kappa )=-\text{log}\;(\frac{\lambda (1-\text{sin}\;[\frac{\pi }{2}(1-e^{-\alpha t^{\delta }})])}{\lambda -\text{sin}\;[\frac{\pi }{2}(1-e^{-\alpha t^{\delta }})]}).$$

**Fig 2:**
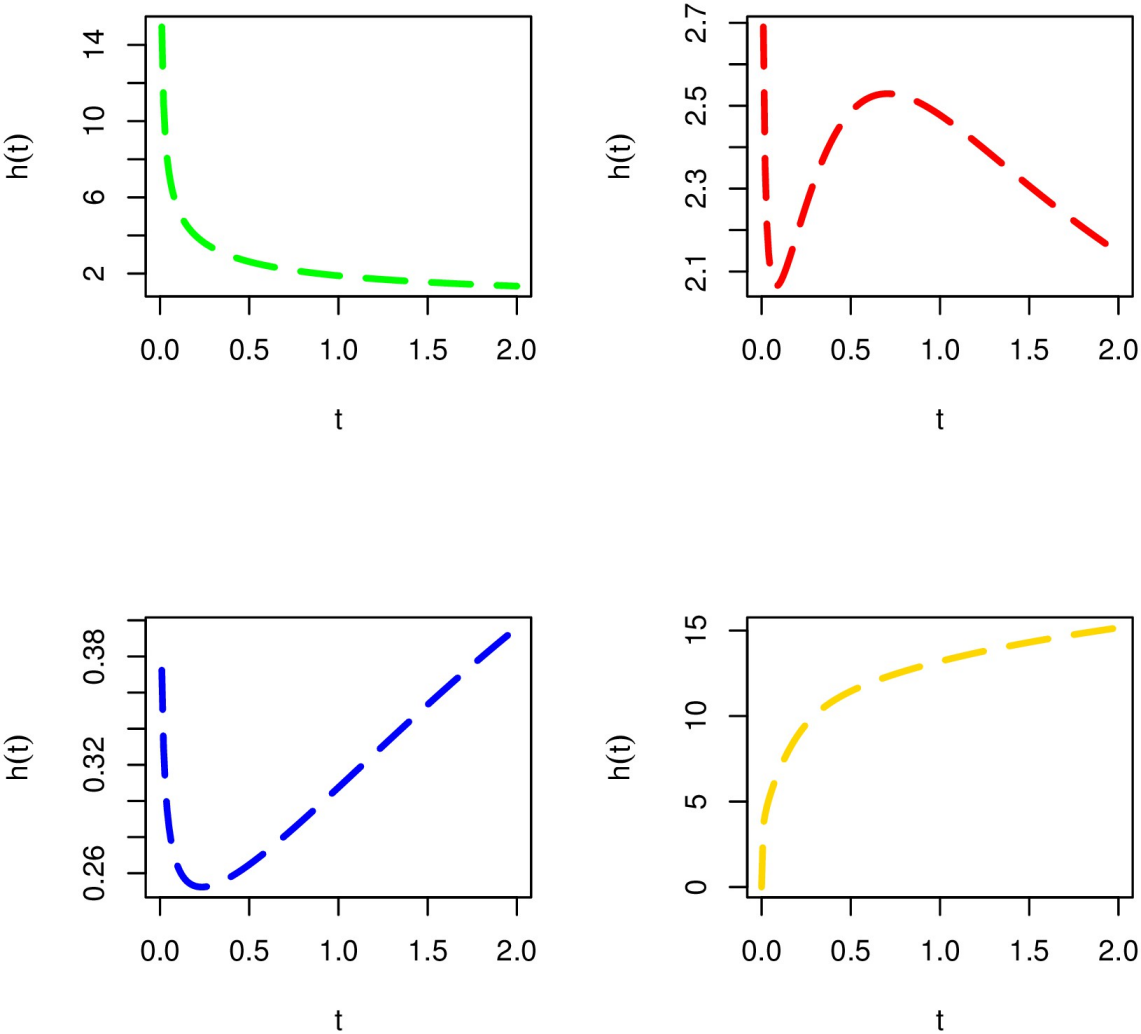
Visual behaviours of *h*(*t*; λ, *κ*) of the NTS-Weibull distribution.

Fig 2 provides different visual behaviors of *h*(*t*; λ, ***κ***) of the NTS-Weibull model. The plots of *h*(*t*; λ, ***κ***) of the NTS-Weibull model are sketched for (*i*) *δ* = 0.5, *α* = 1.9, λ = 9.4 (green line), (*ii*) *δ* = 0.7, *α* = 1.9, λ = 1.4 (red line), (*iii*) *δ* = 0.8, *α* = 0.5, λ = 1.4 (blue line), and (*iv*) *δ* = 1.2, *α* = 5.5, λ = 7.3 (gold line).

Fig 2 shows that *h*(*t*; λ, ***κ***) of the NTS-Weibull distribution has four possible shapes including (*i*) decreasing (green line), (*ii*) decreasing-increasing-decreasing (red line), (*iii*) bathtub (blue line), and (*iv*) increasing (gold line).

Besides, the certain advantages of the NTS-Weibull distribution such as (*i*) the best fitting capability to the healthcare data, (*ii*) different monotonic and non-monotonic shapes of HF, and (*iii*) a best novel control chart, the NTS-Weibull distribution has also certain limitations. The limitations of the NTS-Weibull distribution include

- As the CDF of the NTS-Weibull distribution is not explicit form, therefore, we need to apply an iteration method to generate random numbers.
- Due to the complication form of *f*(*t*; λ, ***κ***) of the NTS-Weibull distribution, more computational efforts are needed to obtain its distributional properties.
- Since, the NTS-Weibull distribution is continuous type distribution, therefore, it cannot be applied to the discrete type of data sets.

## 3 Distributional properties

Here, we derive/calculate some distributional properties of the NTS-*G* family. These distributional properties include the (*i*) quantile function (QF), (*ii*) *r*^*th*^ moment, (*iii*) regular varying tail (RVT) property, and (*iv*) regular variational result (RVR).

### 3.1 The quantile function

The QF (also called the inverse CDF) plays an effective role to generate random numbers for simulation study (SS). Let *T* has the NTS-*G* distributions, then, the QF of *T*, say *t*_*q*_, is given by

$$t_{q}=G^{-1}(\frac{2}{\pi }\;{\text{sin}}^{-1}(\frac{u\lambda }{\lambda -1+u})),$$

where *u* ∈ [0, 1].

### 3.2 The *r*^*th*^ moment

The *r*^*th*^ moment is a useful tools in the statistical literature. It helps to derive the basic summary measures (basic properties) of a probability distributions. These properties include the (*i*) mean expressed by *μ*_1_, (*ii*) variance denoted by *μ*_2_, skewness may be expressed by *γ*_1_, and (*iv*) kurtosis may be denoted by *γ*_2_.

Let *T* follows the NTS-*G* distributions, then, the *r*^*th*^ moment of *T*, say $\mu_{r}^{\prime }$, is obtained as

$$\begin{array}{c} \mu_{r}^{\prime }=\int_{-\infty }^{\infty }t^{r}f(t;\lambda ,\kappa )dt. \end{array}$$ (8)

Using Eq (4) in Eq (8), we obtain

$$\mu_{r}^{\prime }=\int_{-\infty }^{\infty }t^{r}\frac{\pi \lambda \;(\lambda -1)\;g\;(t;\;\kappa )\;\text{cos}\;[\frac{\pi }{2}G(t;\;\kappa )]}{2(\lambda -\text{sin}\;[\frac{\pi }{2}G(t;\;\kappa )]{)}^{2}}dt,$$

$$\mu_{r}^{\prime }=\frac{\pi (\lambda -1)}{2}\sum_{i=1}^{\infty }\frac{i}{\lambda^{i-1}}\int_{-\infty }^{\infty }t^{r}g(t;\;\kappa )\;\text{cos}\;[\frac{\pi }{2}G(t;\;\kappa )](\text{sin}\;[\frac{\pi }{2}G\;(t;\;\kappa )]{)}^{i}dt,$$

$$\mu_{r}^{\prime }=\frac{\pi (\lambda -1)}{2}\sum_{i=1}^{\infty }\frac{i}{\lambda^{i-1}}\int_{-\infty }^{\infty }t^{r}g(t;\;\kappa )\;\text{cos}\;[\frac{\pi }{2}G\;(t;\;\kappa )](\text{sin}\;[\frac{\pi }{2}G\;(t;\;\kappa )]{)}^{i}dt,$$

$$\mu_{r}^{\prime }=\frac{\pi (\lambda -1)}{2}\sum_{i=1}^{\infty }\frac{i(i+1)}{\lambda^{i-1}(i+1)}\int_{-\infty }^{\infty }t^{r}g(t;\;\kappa )\;\text{cos}\;[\frac{\pi }{2}G\;(t;\;\kappa )](\text{sin}\;[\frac{\pi }{2}G\;(t;\;\kappa )]{)}^{(i+1)-1}dt,$$

$$\mu_{r}^{\prime }=(\lambda -1)\sum_{i=1}^{\infty }\frac{i}{\lambda^{i-1}(i+1)}\int_{-\infty }^{\infty }t^{r}m(t;\;\kappa )dt,$$

where

$$\begin{array}{c} m(t;\;\kappa )=\frac{\pi (i+1)}{2}g(t;\;\kappa )\;\text{cos}\;[\frac{\pi }{2}G(t;\;\kappa )](\text{sin}\;[\frac{\pi }{2}G(t;\;\kappa )]{)}^{(i+1)-1}. \end{array}$$ (9)

From the expression of *m*(*t*; ***κ***) in Eq (9), we can see that *m*(*t*; ***κ***) is the exponentiated version of the SS transformation method with exponentiated parameter (*i* + 1).

### 3.3 The HT property

The RVT behavior is a prominent statistical property to identify the HT probability distributions. Here, we obtain the RVT RVT behavior of the NTS-*G* distributions.

According to Seneta [15], using the SF of a probability distribution, we have

**Theorem**: If *F*(*t*; ***κ***) is the SF of a RVT distribution, then *F*(*t*; λ, ***κ***) is also a RVT distribution.

**Proof**: Let assume ${\text{lim}}_{t\to \infty }\frac{F(\beta t;\;\kappa )}{F(t;\;\kappa )}=f(\beta )$ is non-zero and finite ∀ *β* > 0. From Eq (5), we obtain

$$\frac{S(\beta t;\lambda ,\kappa )}{S(t;\lambda ,\kappa )}=\frac{\lambda (1-\text{sin}\;[\frac{\pi }{2}G(\beta t;\;\kappa )])}{\lambda -\text{sin}\;[\frac{\pi }{2}G(\beta t;\;\kappa )]}\times \frac{\lambda -\text{sin}\;[\frac{\pi }{2}G(t;\;\kappa )]}{\lambda (1-\text{sin}\;[\frac{\pi }{2}G(t;\;\kappa )])},$$

$$\begin{array}{c} \frac{S(\beta t;\lambda ,\kappa )}{S(t;\lambda ,\kappa )}=\frac{\lambda (1-\text{sin}\;[\frac{\pi }{2}G(\beta t;\;\kappa )])}{\lambda (1-\text{sin}\;[\frac{\pi }{2}G(t;\;\kappa )])}\times \frac{\lambda -\text{sin}\;[\frac{\pi }{2}G(t;\;\kappa )]}{\lambda -\text{sin}\;[\frac{\pi }{2}G(\beta t;\;\kappa )]}. \end{array}$$ (10)

As from Eq (1), we have $F(t;\;\kappa )=\text{sin}\;[\frac{\pi }{2}G(t;\;\kappa )]$. Thus, from Eq (10), we get

$$\begin{array}{c} \frac{S(\beta t;\lambda ,\kappa )}{S(t;\lambda ,\kappa )}=\frac{\left[1-F(\beta t;\;\kappa )\right]}{\left[1-F(t;\;\kappa )\right]}\times \frac{\lambda -\text{sin}\;[\frac{\pi }{2}G(t;\;\kappa )]}{\lambda -\text{sin}\;[\frac{\pi }{2}G(\beta t;\;\kappa )]}. \end{array}$$ (11)

Applying lim_*t*→∞_ to Eq (11), we get

$$\underset{t\to \infty }{\text{lim}}\frac{S(\beta t;\lambda ,\kappa )}{S(t;\lambda ,\kappa )}=\underset{t\to \infty }{\text{lim}}\frac{\left[1-F(\beta t;\;\kappa )\right]}{\left[1-F(t;\;\kappa )\right]}\times \frac{\lambda -\text{sin}\;[\frac{\pi }{2}G(t;\;\kappa )]}{\lambda -\text{sin}\;[\frac{\pi }{2}G(\beta t;\;\kappa )]},$$

$$\begin{array}{c} \underset{t\to \infty }{\text{lim}}\frac{S(\beta t;\lambda ,\kappa )}{S(t;\lambda ,\kappa )}=f(\beta )\times \underset{t\to \infty }{\text{lim}}\frac{\lambda -\text{sin}\;[\frac{\pi }{2}G(t;\;\kappa )]}{\lambda -\text{sin}\;[\frac{\pi }{2}G(\beta t;\;\kappa )]}. \end{array}$$ (12)

Since, the expression *G*(*t*; ***κ***) is a valid CDF. Therefore, we have lim_*t*→∞_*G*(*t*; ***κ***) = 1 or *G*(∞; ***κ***) = 1. Thus, from Eq (12), we get

$$\underset{t\to \infty }{\text{lim}}\frac{S(\beta t;\lambda ,\kappa )}{S(t;\lambda ,\kappa )}=f(\beta )\times \frac{\lambda -\text{sin}\;[\frac{\pi }{2}G(\infty ;\kappa )]}{\lambda -\text{sin}\;[\frac{\pi }{2}G(\beta \infty ;\kappa )]},$$

$$\underset{t\to \infty }{\text{lim}}\frac{S(\beta t;\lambda ,\kappa )}{S(t;\lambda ,\kappa )}=f(\beta )\times \frac{\lambda -\text{sin}\;[\frac{\pi }{2}]}{\lambda -\text{sin}\;[\frac{\pi }{2}]},$$

$$\begin{array}{c} \underset{t\to \infty }{\text{lim}}\frac{S(\beta t;\lambda ,\kappa )}{S(t;\lambda ,\kappa )}=f(\beta ). \end{array}$$ (13)

Since, *F*(*t*) is non-zero and finite for ∀ *β* > 0, therefore, the expression in Eq (13) confirms that *S*(*t*; λ, ***κ***) is the SF of a RVT distribution.

### 3.4 The RVR

In this section, we discuss the RVR of the NTS-*G* distributions. Suppose *T* obeys the power law property, then we have

$$\bar{F}(t;\;\kappa )=1-F(t;\;\kappa )=P(T>t)\approx t^{\beta }.$$

Using the Karamata’s characterization, we can write the SF *S*(*t*; λ, ***κ***) as follows

$$S(t;\lambda ,\kappa )=t^{\beta }L(t),$$

where *L*(*t*) represents the slowly varying function (SVF). From Eq (5), we have

$$S(t;\lambda ,\kappa )=\frac{\lambda (1-\text{sin}\;[\frac{\pi }{2}G(t;\;\kappa )])}{\lambda -\text{sin}\;[\frac{\pi }{2}G(t;\;\kappa )]},$$

or

$$\begin{array}{c} S(t;\lambda ,\kappa )=\frac{\lambda (1-F(t;\;\kappa ))}{\lambda -\text{sin}\;[\frac{\pi }{2}G(t;\;\kappa )]}. \end{array}$$ (14)

Since 1 − *F*(*t*; ***κ***) ≈ *t*^*β*^, so, we from Eq (14), we get

$$\begin{array}{c} S(t;\lambda ,\kappa )=t^{\beta }L(t), \end{array}$$ (15)

where *L*(*t*) in Eq (15), is given by $\frac{\lambda }{\lambda -\text{sin}\;[\frac{\pi }{2}G(t;\;\kappa )]}$. The function *L*(*t*) will be a SVF, if we could prove

$$\underset{t\to \infty }{\text{lim}}\frac{L(\beta t)}{L(t)}=1.$$

Now consider

$$\frac{L(\beta t)}{L(t)}=\frac{\frac{\lambda }{\lambda -\text{sin}\;[\frac{\pi }{2}G(\beta t;\;\kappa )]}}{\frac{\lambda }{\lambda -\text{sin}\;[\frac{\pi }{2}G(t;\;\kappa )]}},$$

$$\frac{L(\beta t)}{L(t)}=\frac{\lambda -\text{sin}\;[\frac{\pi }{2}G(t;\;\kappa )]}{\lambda -\text{sin}\;[\frac{\pi }{2}G(\beta t;\;\kappa )]},$$

$$\underset{t\to \infty }{\text{lim}}\frac{L(\beta t)}{L(t)}=\underset{t\to \infty }{\text{lim}}\frac{\lambda -\text{sin}\;[\frac{\pi }{2}G(t;\;\kappa )]}{\lambda -\text{sin}\;[\frac{\pi }{2}G(\beta t;\;\kappa )]},$$

$$\underset{t\to \infty }{\text{lim}}\frac{L(\beta t)}{L(t)}=\frac{\lambda -\text{sin}\;[\frac{\pi }{2}G(\infty ;\kappa )]}{\lambda -\text{sin}\;[\frac{\pi }{2}G(\beta .\infty ;\kappa )]},$$

$$\underset{t\to \infty }{\text{lim}}\frac{L(\beta t)}{L(t)}=\frac{\lambda -1}{\lambda -1},$$

$$\underset{t\to \infty }{\text{lim}}\frac{L(\beta t)}{L(t)}=1.$$

Thus, *L*(*t*) is a SVF.

## 4 Estimation and simulation

In the existing literature, numerous approaches have been suggested and implemented for estimating the parameters of the probability distributions. Among them, the maximum likelihood method is the most frequently implemented for estimating the parameters using some observed data. For more detailed information about the maximum likelihood method, we refer to Fisher [16] and Aldrich [17].

This section offers the implementation of the maximum likelihood method for estimating the parameters (λ, ***κ***) of NTS-*G* distributions. In this section, after deriving the estimators $\left(\hat{\lambda },\hat{\kappa }\right)$ of (λ, ***κ***), we provide a SS to demonstrate the behaviors of $\hat{\lambda }$ and $\hat{\kappa }$.

### 4.1 Estimation

Let take a random samples of size *n*, say *T*_1_, *T*_2_, …, *T*_*n*_, from the PDF *f*(*t*; λ, ***κ***) of the NTS-*G* distribution given in Eq (4). Corresponding to *f*(*t*; λ, ***κ***), the likelihood function (LF), say *ϕ*(λ, ***κ***|*t*_1_, *t*_2_, …, *t*_*n*_), is given by

$$\begin{array}{c} \phi (\lambda ,\kappa |t_{1},t_{2},\ldots ,t_{n})=\prod_{i=1}^{n}f(t_{i};\lambda ,\kappa ). \end{array}$$ (16)

Using Eq (4) in Eq (16), we obtain

$$\begin{array}{c} \phi (\lambda ,\kappa |t_{1},t_{2},\ldots ,t_{n})=\prod_{i=1}^{n}\frac{\pi \lambda (\lambda -1)g(t_{i};\kappa )\;\text{cos}\;[\frac{\pi }{2}G(t_{i};\kappa )]}{2(\lambda -\text{sin}\;[\frac{\pi }{2}G(t_{i};\kappa )]{)}^{2}}. \end{array}$$ (17)

Corresponding to *ϕ*(λ, ***κ***|*t*_1_, *t*_2_, …, *t*_*n*_) in Eq (17), the log LF, say log(*ϕ*(λ, ***κ***|*t*_1_, *t*_2_, …, *t*_*n*_)) is given by

$$\begin{array}{cc} \text{log}\;(\phi (\lambda ,\kappa |t_{1},t_{2},\ldots ,t_{n})) & =n\;\text{log}\;\left(\lambda \right)+n\;\text{log}\;(\lambda -1)+\sum_{i=1}^{n}\;\text{log}\;g(t_{i};\kappa )\\ & +n\;\text{log}\;\left(\pi \right)-n\;\text{log}\;\left(2\right)+\sum_{i=1}^{n}\;\text{log}\;\text{cos}\;[\frac{\pi }{2}G(t_{i};\kappa )]\\ & -2\sum_{i=1}^{n}\;\text{log}\;(\lambda -\text{sin}\;[\frac{\pi }{2}G(t_{i};\kappa )]). \end{array}$$ (18)

Corresponding to log(*ϕ*(λ, ***κ***|*t*_1_, *t*_2_, …, *t*_*n*_)) in Eq (18), the partial derivatives are, respectively, given by

$$\frac{\partial }{\partial \lambda }\text{log}\;(\phi (\lambda ,\kappa |t_{1},t_{2},\ldots ,t_{n}))=\frac{n}{\left(\lambda -1\right)}+\frac{n}{\lambda }-\sum_{i=1}^{n}\frac{2}{\left(\lambda -\text{sin}\;[\frac{\pi }{2}G(t_{i};\kappa )]\right)}\;,$$

and

$$\begin{array}{cc} \frac{\partial }{\partial \kappa }\text{log}\;(\phi (\lambda ,\kappa |t_{1},t_{2},\ldots ,t_{n})) & =\sum_{i=1}^{n}\frac{\frac{\partial }{\partial \kappa }g(t_{i};\kappa )}{g(t_{i};\kappa )}-\frac{\pi }{2}\sum_{i=1}^{n}\;g\;(t_{i};\kappa )\text{tan}[\frac{\pi }{2}G(t_{i};\kappa )]\\ & +\pi \sum_{i=1}^{n}\frac{g(t_{i};\kappa )\;\text{cos}\;[\frac{\pi }{2}G(t_{i};\kappa )]}{\left(\lambda -\text{sin}\;[\frac{\pi }{2}G(t_{i};\kappa )]\right)}\;. \end{array}$$

By solving $\frac{\partial }{\partial \lambda }\text{log}\;(\phi (\lambda ,\kappa |t_{1},t_{2},\ldots ,t_{n}))=0$ and $\frac{\partial }{\partial \kappa }\text{log}\;(\phi (\lambda ,\kappa |t_{1},t_{2},\ldots ,t_{n}))=0,$ we get the estimators $\left(\hat{\lambda },\hat{\kappa }\right)$ of the parameters (λ, ***κ***).

As we can see that the MLEs of the NST-Weibull distribution are not explicit forms. Therefore, we use an iteration method with the help of a computer software to find the exact values of the MLEs of the NST-Weibull distribution. To show the uniqueness of ${\hat{\delta }}_{MLE}$, ${\hat{\alpha }}_{MLE}$, and ${\hat{\lambda }}_{MLE},$ the plots for the profiles of the log-likelihood function of *δ*, *α*, and λ are obtained; see Fig 7.

### 4.2 Simulation

This section offers the demonstration of $\hat{\lambda }$ and $\hat{\kappa }$ by conducting a brief SS using the Monte Carlo simulation method (Ulam and von Neumann [18]). The demonstration of $\hat{\lambda }$ and $\hat{\kappa }$ is done by taking random samples of size *n* = 25, 50, …, 500 from the NST-Weibull distribution with PDF *f*(*t*; λ, ***κ***). The replications of the SS are made *N* = 500 times.

The random samples from PDF *f*(*t*; λ, ***κ***) are chosen using the inverse CDF function. Let *T* has the NST-Weibull distribution, then, its inverse CDF is given by

$$\begin{array}{c} t=(-\frac{1}{\alpha }\;\text{log}\;[1-\frac{2}{\pi }\;{\text{sin}}^{-1}\;(\frac{\lambda u}{\lambda -1+u})]{)}^{1/\delta }, \end{array}$$ (19)

where *u* ∈ (0, 1).

The random samples are chosen for three sets of parameters such as (*i*) *δ* = 0.8, *α* = 1.0, λ = 1.4, (*ii*) *δ* = 0.4, *α* = 0.7, λ = 1.5, and (*iii*) *δ* = 1.4, *α* = 1.5, λ = 1.3.

Two evaluation criteria are selected to judge the behaviors of $\hat{\lambda }$ and $\hat{\kappa }$. These evaluation criteria include

- Mean square error (MSE), obtained as
  $$MSE(\hat{\lambda })=\frac{1}{N}\sum_{i=1}^{N}(\hat{\lambda }-\lambda {)}^{2}.$$
- Bias, obtained as
  $$Bias(\hat{\lambda })=\frac{1}{N}\sum_{i=1}^{N}(\hat{\lambda }-\lambda ).$$

The numerical results of the SS of the NST-Weibull distribution are obtained using R-language software with method=L-BFGS-B.

**Fig 3:**
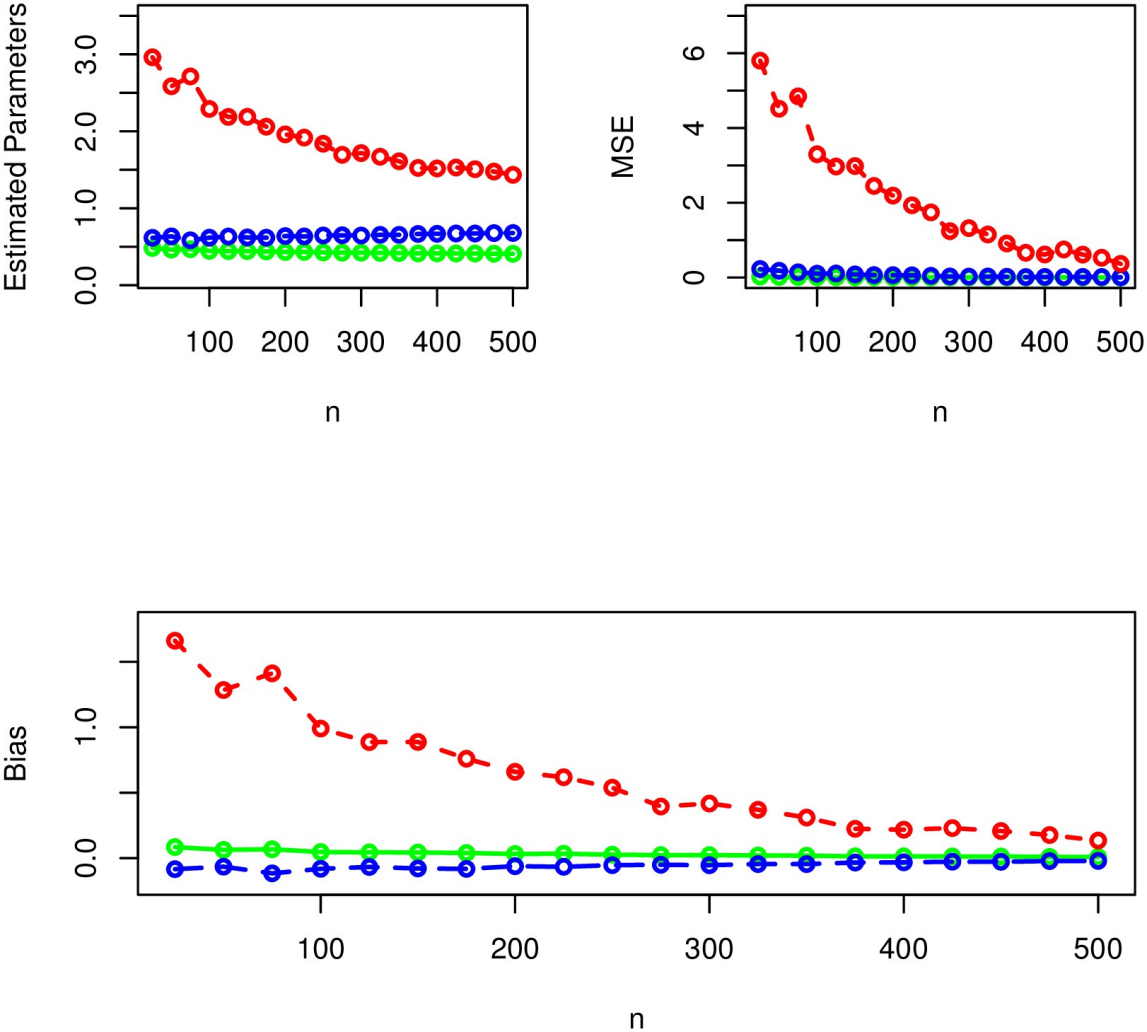
The graphical illustration of the simulation results using *δ* = 0.8 (green curve), *α* = 1.0 (blue curve), and λ = 1.4 (red curve).

**Fig 4:**
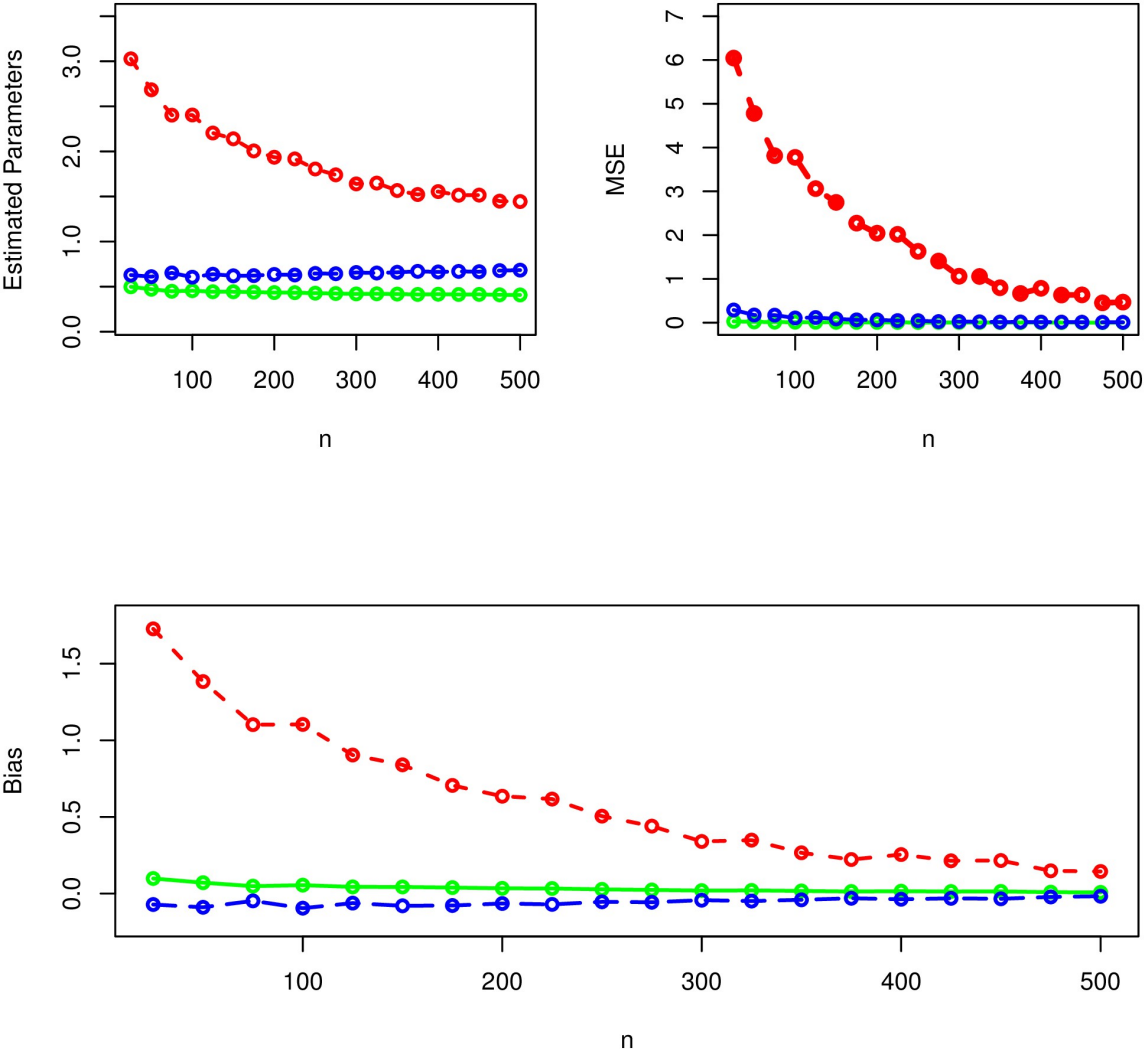
The graphical illustration of the simulation results using *δ* = 0.4 (green curve), *α* = 0.7 (blue curve), and λ = 1.5 (red curve).

**Fig 5:**
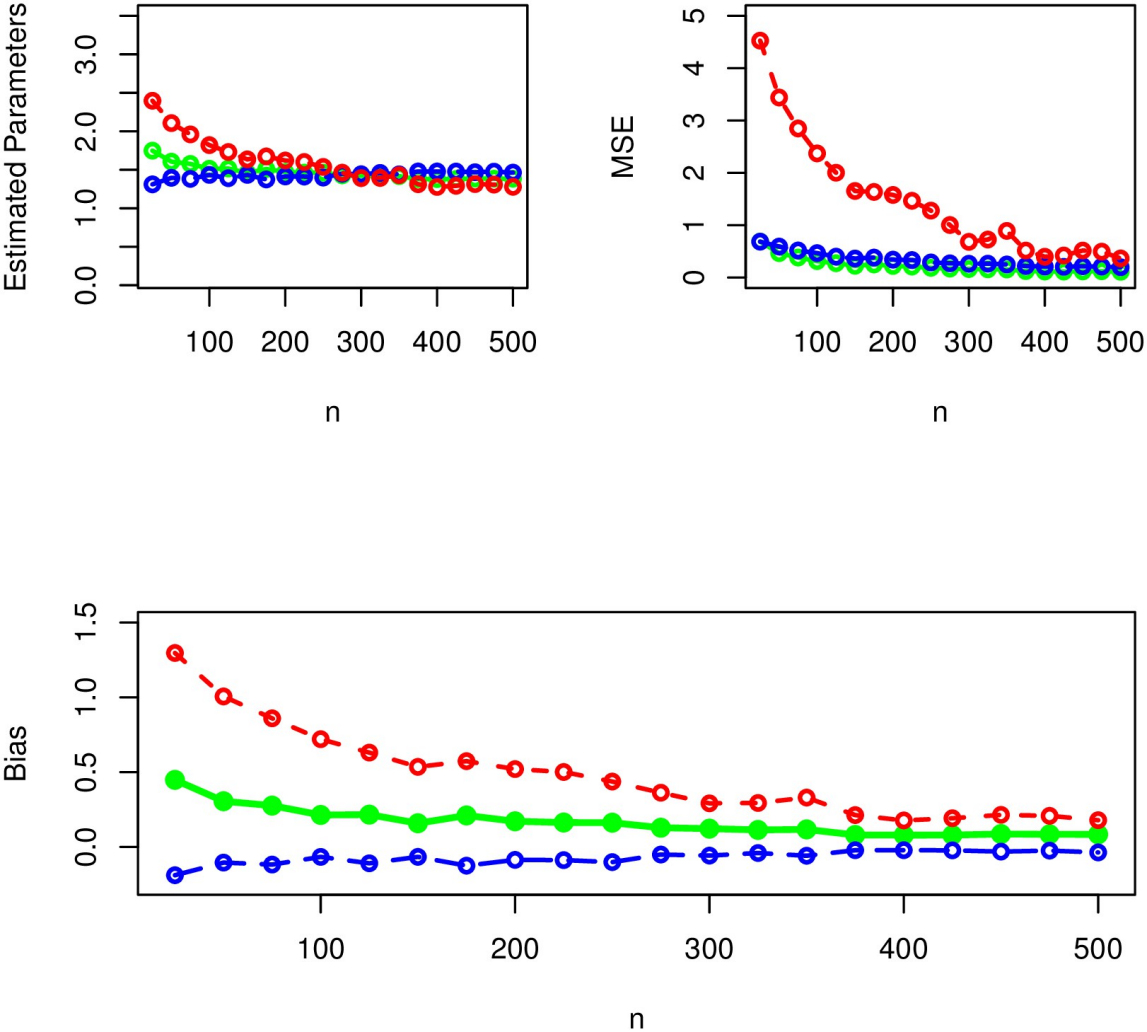
The graphical illustration of the simulation results using *δ* = 1.4 (green curve), *α* = 1.5 (blue curve), and λ = 1.3 (red curve).

Corresponding to *δ* = 0.8, *α* = 1.0, λ = 1.4, the results of the SS of the NST-Weibull distribution are given in Table 1 and presented visually in Fig 3. For the second combination of parameter values (i.e., for *δ* = 0.4*α* = 0.7, λ = 1.5,) the results of the SS of the NST-Weibull model are reported numerically in Table 2 and provided visually in Fig 4. Whereas, for the third combination of parameter values (i.e., for *δ* = 1.4, *α* = 1.5, λ = 1.3.) the simulation results are reported obtained in Table 3 and Fig 5.

**Table 1:** The simulation results using *δ* = 0.8, *α* = 1.0, and λ = 1.4.

*n*	Parameters	MLEs	MSEs	Biases
25	*δ*	0.9967571	0.15115751	0.19675709
	*α*	0.9258497	0.43601030	-0.07415027
	λ	2.7014390	5.32812360	1.50143890
50	*δ*	0.9502273	0.10541820	0.15022727
	*α*	0.9211697	0.35914720	-0.07883033
	λ	2.5398550	4.72918770	1.33985520
75	*δ*	0.9079239	0.08876999	0.10792388
	*α*	0.9907042	0.34370100	-0.00929578
	λ	2.1867230	3.38336300	0.98672340
100	*δ*	0.9021991	0.08189514	0.10219907
	*α*	0.9725867	0.29718490	-0.02741325
	λ	2.1644780	3.26478280	0.96447780
150	*δ*	0.8646130	0.05843910	0.06461299
	*α*	1.0070923	0.26047690	0.00709228
	λ	1.9577740	2.47174710	0.75777360
200	*δ*	0.8571090	0.04858964	0.05710900
	*α*	0.9993452	0.22130800	-0.00065478
	λ	1.7750340	1.75068510	0.57503380
300	*δ*	0.8509225	0.04026877	0.05092246
	*α*	0.9857050	0.16847270	-0.01429501
	λ	1.7307060	1.65020210	0.53070640
400	*δ*	0.8466439	0.03318751	0.04664392
	*α*	0.9820023	0.13594520	-0.01799770
	λ	1.5631160	0.97260570	0.36311590
500	*δ*	0.8185358	0.02649178	0.03853575
	*α*	0.9954864	0.11729050	-0.01451361
	λ	1.4264420	0.61902910	0.26644220

**Table 2:** The simulation results using *δ* = 0.4, *α* = 0.7, and λ = 1.5.

*n*	Parameters	MLEs	MSEs	Biases
25	*δ*	0.4979499	0.03042961	0.09794994
	*α*	0.6055558	0.25338112	-0.09444419
	λ	3.0316110	6.15622390	1.73161070
50	*δ*	0.4748023	0.01861901	0.07480234
	*α*	0.6025582	0.17729227	-0.09744181
	λ	2.7249290	4.93269800	1.42492870
75	*δ*	0.4561838	0.01349214	0.05618378
	*α*	0.6360188	0.17004565	-0.06398118
	λ	2.4604510	3.97853640	1.16045120
100	*δ*	0.4507182	0.01021601	0.05071818
	*α*	0.6157256	0.10883195	-0.08427440
	λ	2.3330160	3.45556920	1.03301580
150	*δ*	0.4327039	0.00744361	0.03270394
	*α*	0.6543529	0.09803863	-0.04564710
	λ	2.0076750	2.31105970	0.70767510
200	*δ*	0.4343523	0.00608387	0.03435232
	*α*	0.6315204	0.05552535	-0.06847958
	λ	1.9389230	2.10971420	0.63892310
300	*δ*	0.4232683	0.00365137	0.02326825
	*α*	0.6470307	0.02845960	-0.05296935
	λ	1.7248370	1.34524250	0.42483740
400	*δ*	0.4120074	0.00168208	0.01200735
	*α*	0.6729542	0.01012379	-0.02704580
	λ	1.4791590	0.51842220	0.17915940
500	*δ*	0.4096051	0.00131430	0.00960513
	*α*	0.6955928	0.00816434	-0.02440723
	λ	1.4909920	0.34001700	0.17099220

**Table 3:** The simulation results using *δ* = 1.4, *α* = 1.5, and λ = 1.3.

*n*	Parameters	MLEs	MSEs	Biases
25	*δ*	1.6683280	0.5692254	0.36832812
	*α*	1.3797750	0.6757503	-0.12022456
	λ	2.2686190	4.0837925	1.16861880
50	*δ*	1.5823070	0.4249679	0.28230654
	*α*	1.4055240	0.6162897	-0.09447634
	λ	2.0967640	3.4581007	0.99676350
75	*δ*	1.5198370	0.3350578	0.21983661
	*α*	1.4277260	0.5059668	-0.07227434
	λ	1.8306650	2.3539422	0.73066470
100	*δ*	1.5136660	0.2792985	0.21366638
	*α*	1.4098040	0.4233400	-0.09019610
	λ	1.8158940	2.2831423	0.71589450
150	*δ*	1.5137040	0.2792985	0.21370398
	*α*	1.3954920	0.4233400	-0.10450813
	λ	1.8058090	2.2831423	0.70580870
200	*δ*	1.4603490	0.2239394	0.16034885
	*α*	1.4300110	0.3460953	-0.06998858
	λ	1.5778340	1.4036935	0.47783440
300	*δ*	1.4500550	0.1770180	0.15005471
	*α*	1.4103790	0.2781250	-0.08962111
	λ	1.4978730	1.1097107	0.39787300
400	*δ*	1.3920700	0.1175921	0.09206995
	*α*	1.4534310	0.2058914	-0.04656887
	λ	1.3026960	0.4310060	0.20269560
500	*δ*	1.3900730	0.1154781	0.09007337
	*α*	1.4948980	0.2001442	-0.04510220
	λ	1.2951020	0.4113533	0.19510210

## 5 Data modeling

**Fig 6:**
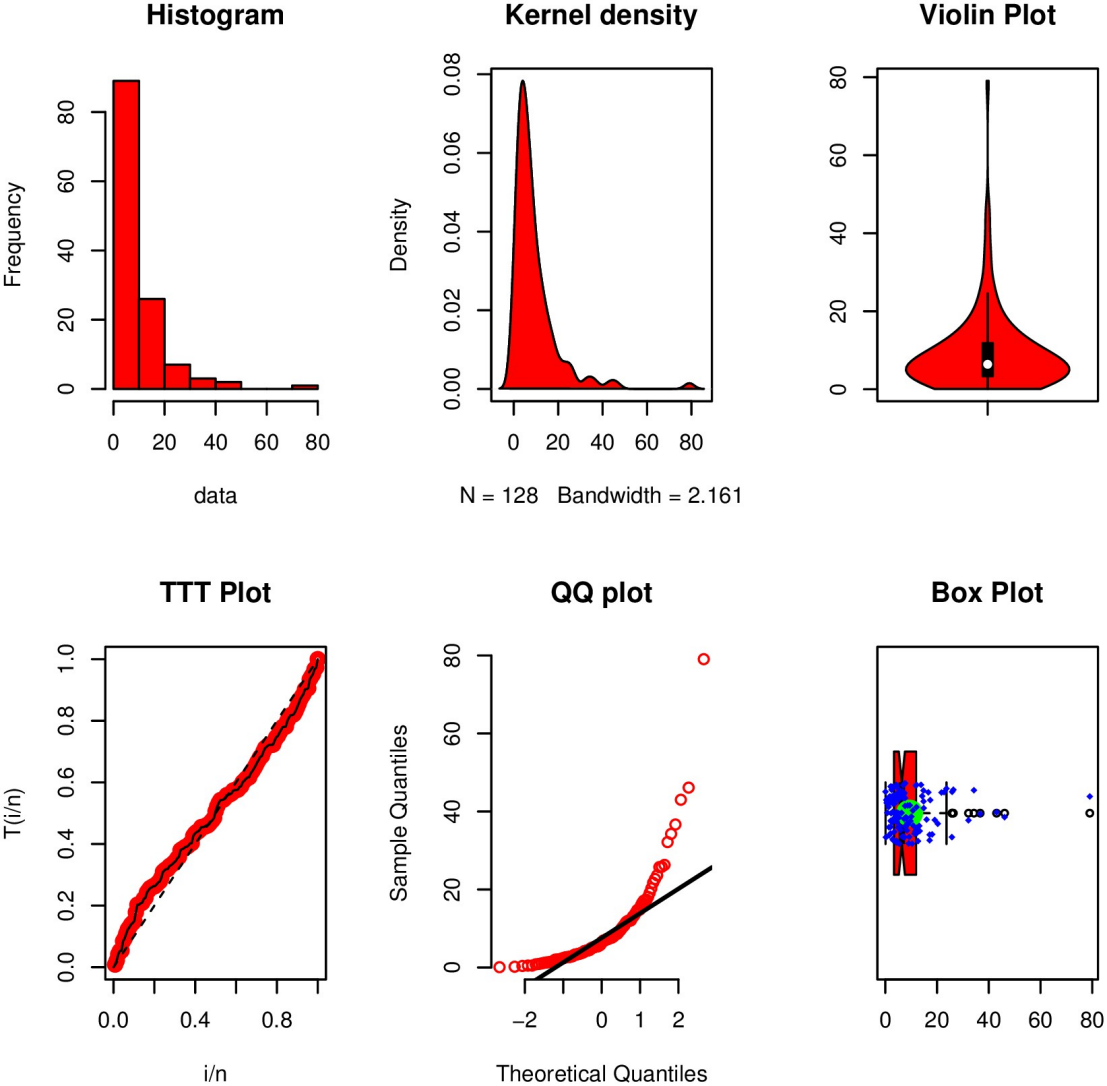
Visual illustration of the survival times data.

The aim of this section is to illustrate the NTS-Weibull distribution in concrete scenarios, especially, survival times data in healthcare and its related areas. We implement the NTS-Weibull distribution to a survival times data set and provide its comparisons with other most famous competing probability models. The survival times data along with the basic measures are presented in Table 4. Furthermore, a visual illustration of the basic plots of the survival times data is displayed in Fig 6.

**Table 4:** The survival times data set and summary statistics (Lee and Wang [19]).

79.05 46.12 43.01 36.66 34.26 32.15 26.31 25.82 25.74 23.63 22.69 21.73 20.28 19.13 18.10					
17.36 17.14 17.12 16.62 15.96 14.83 14.77 14.76 14.24 13.80 13.29 13.11 12.63 12.07 12.03					
12.02 11.98 11.79 11.64 11.25 10.75 9.47 9.22 9.02 8.66 8.65 8.53 8.37 8.26 10.06 9.74 5.17					
7.66 7.63 7.62 7.59 7.39 7.32 7.28 7.26 7.09 6.97 6.94 6.93 6.76 6.54 6.25 5.85 5.71 5.62 1.05					
10.34 7.93 7.87 5.41 5.41 5.34 5.32 5.32 5.09 5.06 4.98 4.87 4.51 4.50 4.40 4.34 4.33 1.19 1.26					
10.66 5.49 4.26 4.23 4.18 3.88 3.82 3.70 3.64 3.57 3.52 3.48 3.36 3.36 3.31 3.25 3.02 2.87 2.83					
2.75 2.69 2.69 2.64 2.62 2.54 2.46 2.26 2.23 2.09 2.07 2.02 2.02 1.76 1.46 1.40 1.35 0.90 0.81					
0.51 0.50 0.40 0.20 0.08					
Smallest	Largest	Mean	Variance	Median	SD
0.080	79.050	9.366	110.425	6.395	10.50833
*Q*_1_	Range	*Q*_3_	Skewness	Kurtosis	*n*
3.348	78.97	11.838	3.286569	18.48308	128

For comparative purposes, the following competing probability models are selected.

- Weibull distribution (Weibull [20])
  $$S(t;\;\kappa )=e^{-\alpha t^{\delta }},\;\;\;\;\;\;\;\;t,\delta ,\alpha >0,$$
- Exponentiated Weibull (E-Weibull) distribution (Mudholkar and Srivastava [21])
  $$S(t;\tau ,\kappa )=1-(1-e^{-\alpha t^{\delta }}{)}^{\tau },\;\;\;\;\;\;\;\;t,\delta ,\alpha ,\tau >0,$$
- Sine inverse Weibull (SI-Weibull) distribution (Souza et al. [6])
  $$S(t;\tau ,\kappa )=1-\text{sin}\;[\frac{\pi }{2}(e^{-\alpha t^{\delta }})],\;\;\;\;\;\;\;\;t,\delta ,\alpha >0,$$
- Sine Weibull (S-Weibull) distribution (Angbing et al. [22])
  $$S(t;\tau ,\kappa )=1-\text{sin}\;[\frac{\pi }{2}(1-e^{-\alpha t^{\delta }})],\;\;\;\;\;\;\;\;t,\delta ,\alpha >0,$$
- New alpha power cosine-Weibull (NAPC-Weibull) distribution (Alghamdi and Abd El-Raouf [23])
  $$S(t;\alpha_{1},\kappa )=\frac{\alpha_{1}-\alpha_{1}^{\text{cos}(\frac{\pi }{2}-\frac{\pi (1-e^{-\alpha t^{\delta }})}{2})}}{\alpha_{1}-1},\;\;\;\;\;\;\;\;t,\delta ,\alpha ,\alpha_{1}>0,\alpha_{1}\neq 1.$$

Among the NTS-Weibull and other selected competing distributions, the decision about the best competing model is made through certain statistical tools. The selection criteria (i.e., statistical tools) are given by

- The Cramér–von Mises (CVM) criterion (Cramér [24])
  The CVM criterion is a useful statistical decisive tool incorporated for comparing two more probability models applied to certain data sets. Generally, a model with the lowest/smallest value of the CVM criterion among the fitted competing probability models is considered the best model. The CVM criterion is obtained as
  $$\sum_{i=1}^{n}{\left[\frac{2i-1}{2n}-G(t_{i})\right]}^{2}+\frac{1}{12n},$$
  where *n* and *t*_*i*_ are, respectively, represent the size of the sample and *i*^*th*^ observation of the data considered for analysis.
- The Anderson–Darling (AD) criterion (Anderson and Darling [25]).
  The AD criterion is another approach for comparing different (at least two) competing probability models. Especially, the AD criterion is most often implemented in scenarios where a family of probability models is being tested. Among the implemented probability models, a model with the lowest value of the AD criterion represents the best suited model. The value of the AD criterion is computed as
  $$-\frac{1}{n}\sum_{i=1}^{n}[\text{log}\{1-G(t_{n-i+1})\}+\text{log}\;G(t_{i})](2i-1)-n.$$
- The Kolmogorov–Smirnov (KS) criterion [(Kolmogorov [26]), (Smirnov [27])]
  In the literature of data modeling, the KS criterion is the most implemented criterion used to find out the best model for a given or certain data set. In the set of given probability models, a model with a smaller value of the KS criterion stands as the best model. The KS criterion value is obtained as
  $$sup_{x}[G_{n}(t)-\hat{G}(t)],$$
  where *G*_*n*_(*t*) and $\hat{G}(t)$ are, respectively, called the estimated CDF and empirical CDF.

All the numerical computation of the real data analysis is carried out using optim()R-language software with SANN method.

**Fig 7:**
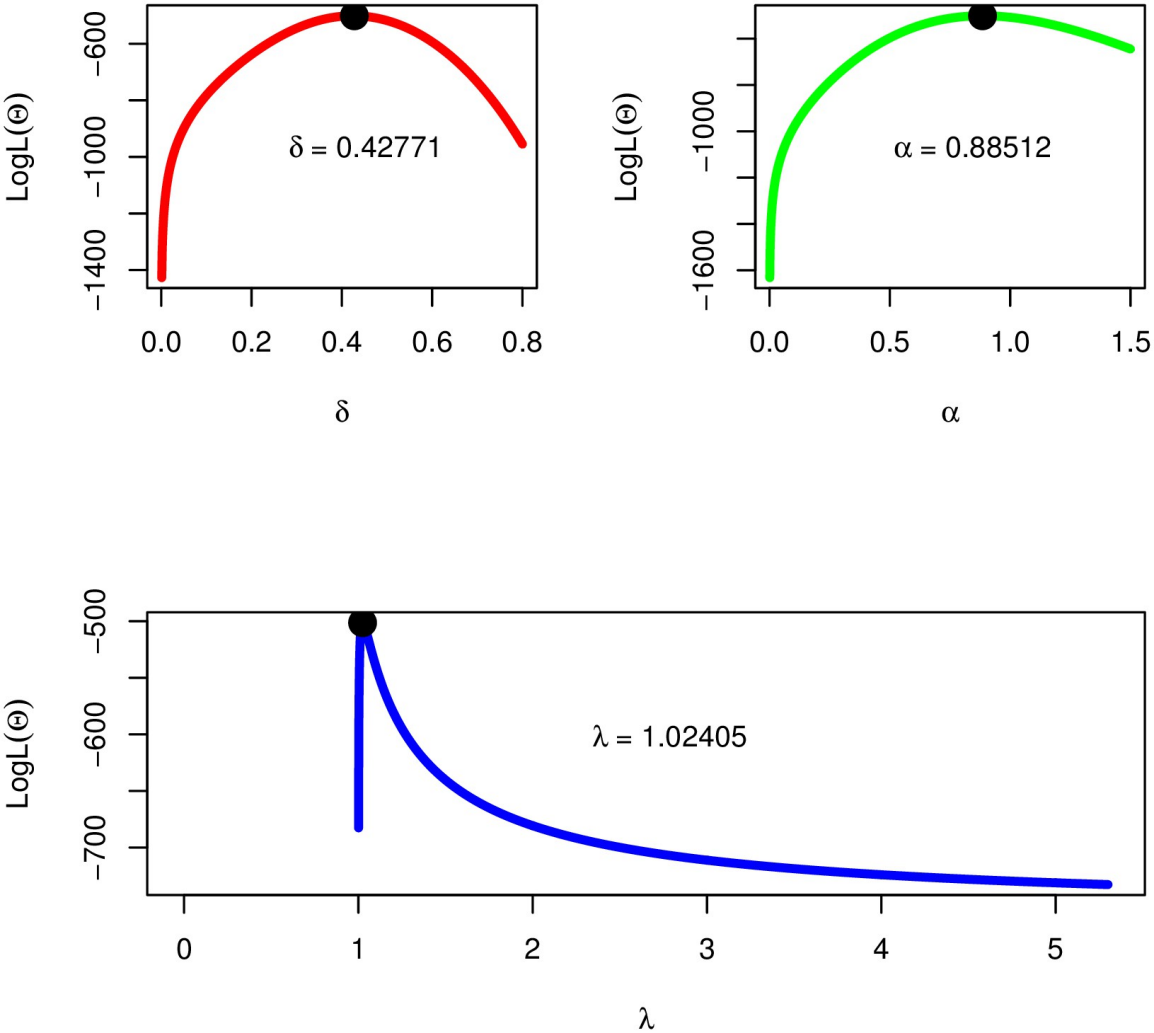
The profiles of the LLF of ${\hat{\delta }}_{MLE},{\hat{\alpha }}_{MLE},$ and ${\hat{\lambda }}_{MLE}$ of the NTS-Weibull distribution using the survival times data.

After analyzing the survival times data, the MLEs (i.e., the numerical values of ${\hat{\delta }}_{MLE},{\hat{\alpha }}_{MLE},{\hat{\lambda }}_{MLE},{\hat{\tau }}_{MLE})$ of competing models are shown in Table 5. Since, the MLEs of the NTS-Weibull are not in closed form. Therefore, to ensure unique solutions of ${\hat{\delta }}_{MLE},{\hat{\alpha }}_{MLE},$ and ${\hat{\lambda }}_{MLE}$ of the NTS-Weibull model, the profiles of the log LF of ${\hat{\delta }}_{MLE},{\hat{\alpha }}_{MLE},$ and ${\hat{\lambda }}_{MLE}$ are presented in Fig 7.

**Table 5:** The values of ${\hat{\delta }}_{MLE},{\hat{\alpha }}_{MLE},{\hat{\lambda }}_{MLE}$, and ${\hat{\tau }}_{MLE}$ of the fitted models for the survival times data.

Model	${\hat{\delta }}_{MLE}$	${\hat{\alpha }}_{MLE}$	${\hat{\lambda }}_{MLE}$	${\hat{\tau }}_{MLE}$	${\hat{\tau }}_{MLE}$
NTS-Weibull	0.42771	0.88512	1.02405	-	-
Weibull	1.05357	0.09165	-	-	-
E-Weibull	0.34230	1.69017	-	14.36856	
NAC-Weibull	0.75268	0.17150	-	-	8.00968
S-Weibull	0.99051	0.06139	-	-	-
SI-Weibull	0.61871	3.10102	-	-	-

Furthermore, the values of the selected criteria and the p-value of the NTS-Weibull distribution are presented in Table 6. According to Table 6, it is obvious that the NTS-Weibull model is the best suitable model for analyzing the survival times data.

**Table 6:** The values of analytical measures of the fitted models for the survival times data.

Model	CVM	AD	KS	p-value
NTS-Weibull	0.10797	0.63338	0.04926	0.91520
Weibull	0.13241	0.79257	0.07429	0.47980
E-Weibull	0.11874	0.80243	0.06968	0.56310
NAC-Weibull	0.12485	0.73786	0.06674	0.61850
S-Weibull	0.14015	0.83398	0.07168	0.52630
SI-Weibull	0.42346	2.68593	0.11046	0.08800

**Fig 8:**
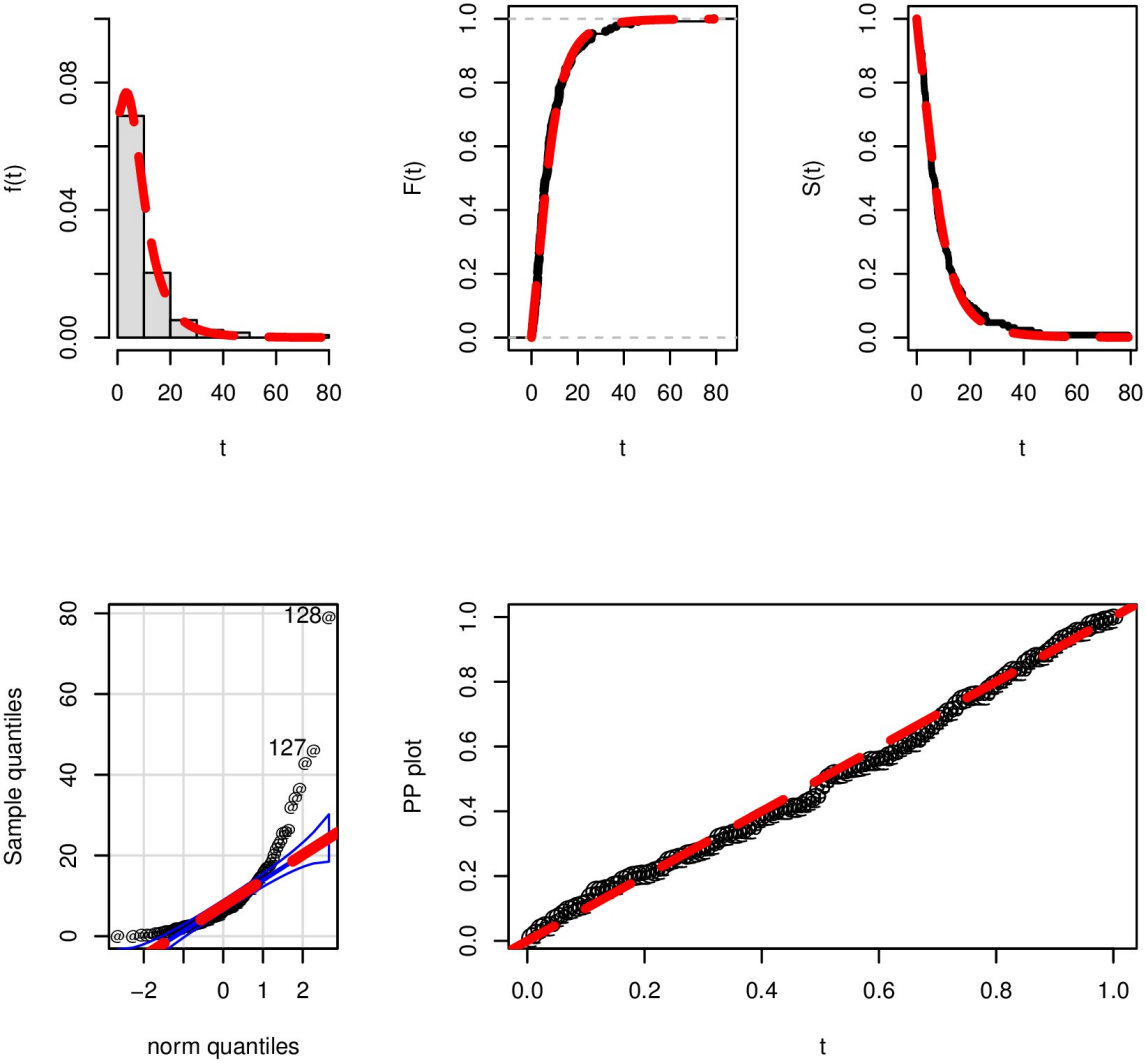
Visual display of the best-fitting ability of the NTS-Weibull distribution.

To support the close-fitting (best-fitting) power of the NTS-Weibull model in Table 6, a visual display of the NTS-Weibull distribution performances is presented in Fig 8. For showing the fitting power of the NTS-Weibull model visually, we consider different graphical approaches such as the (*i*) estimated PDF, (*ii*) estimated CDF, (*iii*) estimated SF (*iv*) probability-probability (PP) plot, and (*v*) quantile-quantile (QQ) plot. The obtained plots in Fig 8 reveal that the NTS-Weibull model fits the survival times data very closely.

## 6 A new attribute control chart based on the truncated life test

To keep the production process under control, various useful control charts are available. One of these charts shows the difference between the lower control limit (LCL) and upper control limit (UCL). There are two well-known types of control charts used in statistical quality control processes, namely, control charts for attributes and control charts for variables. Since it uses quantitative data, the variables control chart offers useful information regarding the procedure and includes minimal sample sizes. Due to its ease of computation, the use of attribute control charts is more versatile than the usage of variable charts. The *np* chart, *u* chart, and *c* chart are a few of the well-known attribute control charts in the literature.

Numerous writers have explored the attribute control chart for the time-shortened life test for different distributions, please refer to Aslam et al. [28], Quinino et al. [29], Adeoti and Ogundipe [30], Adeoti and Rao [31], and Rao and Al-Omari [32]. According to our review of the existing literature, there is no work on attribute control charts based on the probability distributions that are developed using the sine function. In this study, we cover this gap and project a new attribute control chart based on a truncated life test for the NTS-Weibull distribution.

### 6.1 The proposed control chart

On the basis of the time-truncated life testing of Aslam and Jun [33], we project a new *np* control chart that is as follows:

- **Step 1**: Select a size-n simple random sample from the submitted lot and examine the results. The number of failures caused by (*D*) is determined before the experiment begins, *t*_0_ = *τξ*_0_, where *ξ*_0_ is the quality factor and *τ* is a multiplier constant under the assumption that the process is under control.
- **Step 2**: Declare the process to be out of control when *D* > *UCL* or *D* < *LCL* otherwise the process is in-control if *LCL* < *D* < *UCL*.

The percentile of the NST-Weibull distribution is

$$\begin{array}{c} t_{q}=(-\frac{1}{\alpha }\text{log}[1-\frac{2}{\pi }{\text{sin}}^{-1}(\frac{\lambda q}{\lambda -1+q})]{)}^{1/\delta }. \end{array}$$ (20)

Let

$$\alpha =-\frac{-\text{log}[1-\frac{2}{\pi }{\text{sin}}^{-1}(\frac{\lambda q}{\lambda -1+q})]}{t_{q}^{\delta }}\;,$$

or

$$\alpha =\frac{\eta_{q}}{t_{q}^{\delta }}\;,$$

where

$$\eta_{q}=-\text{log}[1-\frac{2}{\pi }{\text{sin}}^{-1}(\frac{\lambda q}{\lambda -1+q})].$$

Using the binomial distribution of defective products with parameters *p*_0_ and *n*, the proposed chart limits are obtained as

$$UCL=np_{0}+k\sqrt{np_{0}(1-p_{0})},$$

and

$$LCL=\text{max}\{0,np_{0}-k\sqrt{np_{0}(1-p_{0})}\},$$

where *p*_0_ is the likelihood that an article will fail testing before time *t*_0_ when the process is thought to be under control, and *k* is the desired chart coefficient. However, once *ξ* = *ξ*_0_ is present, we may state that the process is under control (*i*.*e*., *δ* = *δ*_0_, λ = λ_0_, *α* = *α*_0_).

Let’s assume that the experiment time is *t*_0_ in a time-truncated life span experimentation, *a* is a multiple of the termination ratio and the stated percentile life *t*_*q*0_, i.e., *t*_0_ = *τt*_0_. The likelihood of failure is written as a result of simplification as

$$\begin{array}{c} p_{0}=1-\frac{\lambda (1-\text{sin}\;[\frac{\pi }{2}(1-e^{-\frac{\tau^{\delta }\eta_{q}}{{\left(t_{q}/t_{q0}\right)}^{\delta }}})])}{\lambda -\text{sin}\;[\frac{\pi }{2}(1-e^{-\frac{\tau^{\delta }\eta_{q}}{{\left(t_{q}/t_{q0}\right)}^{\delta }}})]}. \end{array}$$ (21)

When the process is in-control, then the percentile ratio *t*_*q*_/*t*_*q*0_ = 1. Therefore, Eq (21) will be reduced to

$$\begin{array}{c} p_{0}=1-\frac{\lambda (1-\text{sin}\;[\frac{\pi }{2}(1-e^{-\tau^{\delta }\eta_{q}})])}{\lambda -\text{sin}\;[\frac{\pi }{2}(1-e^{-\tau^{\delta }\eta_{q}})]}. \end{array}$$ (22)

Now, we consider the percentile ratio *t*_*q*_/*t*_*q*0_ = *c*, where *c* = 0.1, 0.2, …, 4.0 Then, the probability in Eq (21) became

$$p_{0}=1-\frac{\lambda (1-\text{sin}\;[\frac{\pi }{2}(1-e^{-\eta_{q}{\left(\tau /c\right)}^{\delta }})])}{\lambda -\text{sin}\;[\frac{\pi }{2}(1-e^{-\eta_{q}{\left(\tau /c\right)}^{\delta }})]}.$$

Let $\bar{D}$ be the sample-average failure rate for the subgroups. The following equations can be used to calculate the chart limits for realistic purposes if the value of *p*_0_ is unknown, then

$$UCL=\bar{D}+k\sqrt{\bar{D}(1-\bar{D}/n)},$$

and

$$LCL=\text{max}\{\bar{D}-k\sqrt{\bar{D}(1-\bar{D}/n)}\}.$$

The following factors increase the likelihood that the process can be declared to be under control for the created control chart:

$$p_{IC}^{0}=P\{LCL\leq D\leq UCL|p_{0}\}=\sum_{d=\lfloor LCL\rfloor +1}^{\left\lfloor LCL\right\rfloor }\left(\begin{array}{c} n\\ d \end{array}\right)p_{0}^{d}(1-p_{0}{)}^{n-d}.$$

The average control length (ARL), which is expressed as follows when the process is in control, allows one to evaluate the success of the prepared control chart:

$$ARL_{0}=\frac{1}{1-P_{IC}^{0}}.$$

It is necessary to look into the study of out-of-control in order to assess the effectiveness of the proposed control chart. Assume *p*_1_ as the probability of a failure item occurring before the experiment time *t*_0_ when the process is out of control. As a result, the likelihood that the process is in control even though the declared time ratio is altered to *c* is given by

$$p_{IC}^{0}=P\{LCL\leq D\leq UCL|p_{1}\}=\sum_{d=\lfloor LCL\rfloor +1}^{\left\lfloor LCL\right\rfloor }\left(\begin{array}{c} n\\ d \end{array}\right)p_{1}^{d}(1-p_{1}{)}^{n-d}.$$

Thus, the ARL for the shifted process is obtained as follows

$$\begin{array}{c} ARL_{1}=\frac{1}{1-P_{IC}^{1}}. \end{array}$$ (23)

The following step-by-step procedure can be used to acquire the tables of the developed control chart.

- Find out the ARL value, say *r*_0_ and known parametric values *δ* = *δ*_0_, λ = λ_0_, and *α* = *α*_0_, respectively.
- Determine the chart constants *k*, *τ*, and *n* such that the *ARL*_0_ value is almost equal to *r*_0_, i.e., *ARL*_0_ ≥ *r*_0_.
- Once you have the numbers from Step 2, calculate the *ARL*_1_ using the shift constant *c* based on Eq. (23).

For different values of *δ* = *δ*_0_, λ = λ_0_, *r*_0_ and *n*, control chart’s parameters and *ARL*_1_ are provided in Tables 7–10 for shift values.

**Table 7:** The ARLs of the proposed chart for *δ* = 2.5, λ = 1.5, and *n* = 20.

*r*_0_	200	250	300	370	500
*k*	2.890	3.577	2.940	3.031	3.481
*τ*	0.933	0.774	0.977	0.991	0.819
*c*	*ARL*_1_	*ARL*_1_	*ARL*_1_	*ARL*_1_	*ARL*_1_
0.10	1.00	1.00	1.00	1.00	1.00
0.20	1.00	1.00	1.00	1.00	1.00
0.30	1.00	1.01	1.00	1.00	1.00
0.40	1.04	1.34	1.03	1.07	1.21
0.50	1.41	3.00	1.34	1.64	2.40
0.60	2.99	9.12	2.70	4.05	6.99
0.70	8.62	30.54	7.64	14.03	23.93
0.80	28.87	98.11	26.02	57.34	83.81
0.85	53.56	161.36	49.58	117.54	152.85
0.90	96.53	230.27	94.76	228.60	263.80
0.95	156.53	236.06	177.00	360.33	403.44
1.00	200.10	250.28	302.03	370.92	501.53
1.05	187.96	207.29	245.11	264.73	492.74
1.10	143.35	163.22	197.58	167.10	410.62
1.15	101.74	127.50	129.93	106.07	318.36
1.20	72.17	100.62	106.36	70.10	242.96
1.30	39.20	65.83	79.14	34.84	146.34
1.40	23.79	45.97	53.54	20.00	94.69
1.50	15.81	33.89	32.28	12.80	65.43
1.60	11.29	26.11	21.24	8.91	47.70
1.70	8.52	20.83	14.97	6.62	36.32
1.80	6.72	17.11	11.15	5.17	28.64
1.90	5.50	14.39	8.67	4.21	23.25
2.00	4.62	12.34	6.99	3.53	19.33
3.00	1.89	4.91	2.28	1.53	6.43
4.00	1.39	3.20	1.54	1.20	3.87

**Table 8:** The ARLs of the proposed chart for *δ* = 4.1, λ = 1.3, and *n* = 20.

*r*_0_	200	250	300	370	500
*k*	2.828	2.768	4.124	3.03	3.656
*τ*	0.959	1.097	0.867	0.995	0.888
*c*	*ARL*_1_	*ARL*_1_	*ARL*_1_	*ARL*_1_	*ARL*_1_
0.10	1.00	1.00	1.00	1.00	1.00
0.20	1.00	1.00	1.00	1.00	1.00
0.30	1.00	1.01	1.01	1.00	1.00
0.40	1.01	1.13	1.32	1.02	1.11
0.50	1.24	1.61	3.50	1.33	2.05
0.60	2.42	3.17	14.39	2.87	6.30
0.70	7.19	8.72	67.63	9.74	24.48
0.80	26.78	31.36	163.88	43.07	99.53
0.85	53.18	63.90	183.75	94.43	193.22
0.90	102.25	131.63	207.23	200.79	339.95
0.95	169.87	236.39	226.72	349.49	481.11
1.00	202.30	250.05	301.13	370.50	501.20
1.05	167.30	147.71	213.85	250.84	412.24
1.10	115.85	75.01	155.02	149.06	307.01
1.15	77.94	40.10	115.70	90.43	224.89
1.20	53.76	23.33	88.87	57.85	167.20
1.30	28.55	9.91	56.48	27.64	99.21
1.40	17.26	5.33	38.88	15.57	64.34
1.50	11.53	3.40	28.45	9.91	44.80
1.60	8.31	2.45	21.84	6.91	32.99
1.70	6.35	1.93	17.41	5.17	25.39
1.80	5.07	1.62	14.30	4.07	20.25
1.90	4.20	1.43	12.05	3.35	16.62
2.00	3.59	1.30	10.36	2.85	13.97
3.00	1.63	1.02	4.26	1.37	5.06
4.00	1.27	1.00	2.85	1.13	3.22

**Table 9:** The ARLs of the proposed chart for *δ* = 2.5, λ = 1.5, and *n* = 30.

*r*_0_	200	250	300	370	500
*k*	2.907	2.992	2.973	2.981	3.123
*τ*	0.948	1.105	1.185	0.948	1.170
*c*	*ARL*_1_	*ARL*_1_	*ARL*_1_	*ARL*_1_	*ARL*_1_
0.10	1.00	1.00	1.00	1.00	1.00
0.20	1.00	1.00	1.00	1.00	1.00
0.30	1.00	1.00	1.00	1.00	1.00
0.40	1.00	1.01	1.04	1.00	1.05
0.50	1.08	1.15	1.32	1.18	1.42
0.60	1.71	2.02	2.56	2.34	3.08
0.70	4.41	6.06	7.85	7.84	10.69
0.80	15.87	27.36	34.72	35.86	53.39
0.85	32.29	64.12	80.54	80.98	130.98
0.90	66.54	151.35	189.83	179.48	325.17
0.95	130.15	282.85	357.13	331.77	628.08
1.00	201.32	251.77	300.26	370.67	500.60
1.05	198.09	130.52	141.02	252.96	221.68
1.10	137.72	64.50	64.35	148.52	97.27
1.15	86.83	34.63	32.53	88.80	47.60
1.20	55.65	20.37	18.27	56.04	25.95
1.30	26.11	8.85	7.49	26.13	10.07
1.40	14.42	4.85	4.00	14.43	5.11
1.50	9.03	3.14	2.58	9.03	3.15
1.60	6.22	2.29	1.90	6.22	2.24
1.70	4.61	1.82	1.54	4.61	1.75
1.80	3.61	1.54	1.34	3.61	1.48
1.90	2.96	1.37	1.21	2.96	1.31
2.00	2.51	1.25	1.14	2.51	1.20
3.00	1.24	1.01	1.00	1.24	1.00
4.00	1.07	1.00	1.00	1.07	1.00

**Table 10:** The ARLs of the proposed chart for *δ* = 4.1, λ = 1.3, and *n* = 30.

*r*_0_	200	250	300	370	500
*k*	2.912	2.961	2.971	3.104	3.348
*τ*	0.971	0.924	0.884	0.978	0.859
*c*	*ARL*_1_	*ARL*_1_	*ARL*_1_	*ARL*_1_	*ARL*_1_
0.10	1.00	1.00	1.00	1.00	1.00
0.20	1.00	1.00	1.00	1.00	1.00
0.30	1.00	1.00	1.00	1.00	1.00
0.40	1.00	1.00	1.00	1.00	1.02
0.50	1.03	1.06	1.18	1.04	1.37
0.60	1.39	1.63	2.27	1.53	3.16
0.70	3.27	4.20	6.76	4.12	10.42
0.80	12.17	15.16	25.04	17.74	39.60
0.85	26.07	30.77	49.66	40.93	78.11
0.90	57.69	63.65	97.88	97.45	152.60
0.95	122.93	130.72	184.33	222.27	288.60
1.00	202.07	250.21	301.96	371.73	502.04
1.05	191.38	202.25	274.20	324.40	429.54
1.10	122.81	194.79	239.13	190.59	311.26
1.15	73.04	148.19	216.26	106.96	282.14
1.20	45.13	131.52	183.58	63.36	253.09
1.30	20.37	94.08	97.36	26.84	211.50
1.40	11.11	50.08	57.15	13.94	185.64
1.50	6.96	29.99	36.76	8.41	119.52
1.60	4.83	19.68	25.43	5.66	82.16
1.70	3.63	13.87	18.63	4.14	59.55
1.80	2.89	10.34	14.28	3.23	45.05
1.90	2.41	8.06	11.36	2.65	35.29
2.00	2.08	6.52	9.31	2.25	28.45
3.00	1.16	2.18	3.11	1.18	8.01
4.00	1.04	1.50	2.01	1.05	4.51

Based on computed tables (i.e., Tables 7–10), we observed the following conclusions:

- It is observed that *ARL*_1_ value is in decreasing tendency as the shift value *c* increases.
- From Tables 7 and 8, it is evident that the *ARL*_1_ values are decreases with the increase of parametric values.
- Based on Tables 7–10, it is reasonable that when the sample size *n* increases from 20 to 30, the *ARL*_1_ values are shows decreasing tendency.

### 6.2 An example of the recommended control chart

The created control chart is demonstrated as follows: let us assume that industrial output persists in the NTS-Weibull distribution with parameters *δ* = 2.5, λ = 1.5. Suppose the average product goal lifetime is *ξ*_0_ = 1000 hours and *r*_0_ = 370. Using Eq. (22) the value of *p*_0_ is 0.4367. Also, from Table 9 the chart parameters are *n* = 30, *τ* = 0.948, *k* = 2.981, *LCL* = 5, and *UCL* = 21. Hence, the experiment time *t*_0_ is 948 hours. As a result, the proposed control chart was implemented as follows:

- **Step 1**: Take a basic random sample of 30 people from each category and subject them to a 948-hour assessment of their quality of life. For the period of the experiment, ascertain the number of failed units, let’s say *D*.
- **Step 2**: If 5 ≤ *D* ≤ 21, declare the production process to be under control; if not, declare it to be out of control.

### 6.3 Industrial use

Using the survival times data set given in Section 4 the estimated parameters of NTS-Weibull distribution are $\hat{\delta }=0.42771$, $\hat{\alpha }=0.88512$, and $\hat{\lambda }=1.02405.$ Table 11 provides the ARLs of the proposed control chart. Here, $n=20,\hat{\delta }=0.42771$, $\hat{\lambda }=1.02405$, *τ* = 0.773, and *r*_0_ = 500. The value of *p*_0_ is 0.39527 using Eq (22). The value of *ξ* by Eq (20) is obtained as 6.64895 for the duration of test *t*_0_ = *τξ*_0_ = 0.773×6.64895 = 5.1369.

**Table 11:** The ARLs of the proposed chart for $\hat{\delta }=0.4277,\hat{\lambda }=1.0241$, and *n* = 30.

*r*_0_	200	250	300	370	500
*k*	2.890	2.955	2.941	3.060	3.158
*τ*	0.822	0.719	0.936	0.908	0.773
*c*	*ARL*_1_	*ARL*_1_	*ARL*_1_	*ARL*_1_	*ARL*_1_
0.10	1.00	1.00	1.00	1.00	1.00
0.20	1.00	1.00	1.00	1.00	1.00
0.30	1.00	1.00	1.00	1.00	1.00
0.40	1.00	1.00	1.00	1.00	1.00
0.45	1.00	1.00	1.00	1.00	1.00
0.50	1.00	1.00	1.00	1.00	1.00
0.60	1.01	1.02	1.02	1.02	1.03
0.70	1.37	1.43	1.35	1.43	1.57
0.80	4.25	4.76	4.00	4.73	6.10
0.85	10.50	11.90	9.73	12.17	16.76
0.90	30.57	34.34	28.47	37.45	53.56
0.95	95.53	105.53	94.96	129.17	185.06
1.00	200.14	250.72	301.08	370.86	500.23
1.05	136.28	228.19	287.27	327.56	441.83
1.10	62.65	117.26	177.56	137.89	203.18
1.15	31.44	60.72	76.81	61.32	96.66
1.20	17.90	34.83	38.16	31.49	51.90
1.30	7.78	14.93	13.47	11.72	20.15
1.40	4.44	8.22	6.65	6.00	10.35
1.50	3.02	5.34	4.08	3.78	6.40
1.60	2.30	3.88	2.89	2.73	4.48
1.70	1.89	3.05	2.26	2.16	3.43
1.80	1.64	2.53	1.89	1.82	2.79
1.90	1.48	2.19	1.65	1.60	2.37
2.00	1.37	1.95	1.49	1.46	2.09
3.00	1.06	1.23	1.07	1.07	1.25
4.00	1.02	1.10	1.02	1.02	1.11

**Fig 9:**
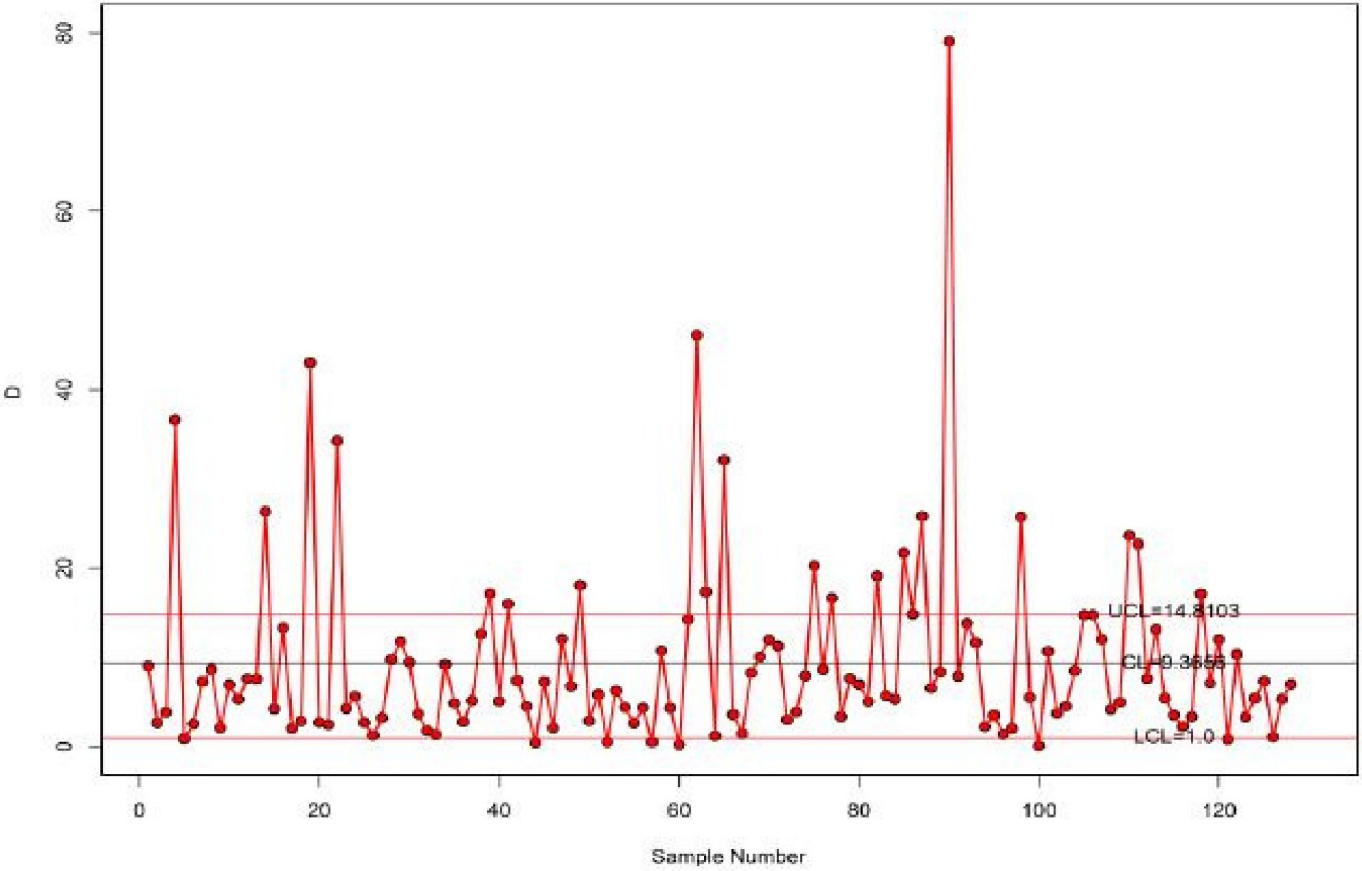
Proposed control chart for survival time data.

The proposed control limits chart has *LCL* = 1 and *UCL* = 14.8103 for the *k* = 3.158 parameter. Fig 9 shows the suggested control chart for the survival time data. It can be seen from Fig 9 that the suggested chart exhibits out-of-control survival time data. As a result, the suggested control chart efficiently detects the quality of the survival time data.

### 6.4 Comparison

The ARL values of the proposed control chart and the existing time truncated life testing attributed control charts for the Weibull distribution given in Adeoti and Rao (2022) are compared. The results of comparisons between the NTS-Weibull distribution and Weibull distribution are displayed in Table 12. To compare the two types of control charts with respect to ARL values at different shift values. It is important to note that a chart having lesser out-of-control ARLs would be considered the better control chart. We noticed based on Table 12 the ARL values of the developed control chart have fever ARL values as compared with the control chart developed for the Weibull distribution. For instance, when *c* = 1.5 the *ARL*_1_ of the developed NTS-Weibull control chart for *n* = 20 is 12.80. Whereas, the *ARL*_1_ for the Weibull distribution is 29.40. Hence we conclude that the proposed chart is speedy to find process changes as compared with the existing control chart established on the Weibull distribution.

**Table 12:** ARLs of attribute control charts for NTS-Weibull and Weibull distributions for *ARL*_0_ = 370 and *n* = 20.

*L*	3.031	2.8495
*τ*	0.991	0.6959
*c*	NTS-Weibull	Weibull
1.0	370.92	370.1
1.1	167.10	438.1
1.2	70.10	193.79
1.3	34.84	89.99
1.4	20.00	48.47
1.5	12.80	29.4
1.6	8.91	19.52
1.7	6.62	13.9
1.8	5.17	10.46
1.9	4.21	8.23
2	3.53	6.69

## 7 Concluding remarks

In this paper, a new method using the trigonometric function for generating updated probability distributions was introduced in this paper. The proposed approach was named a new trigonometric sine-*G* method. The NTS-*G* method was introduced by using the sine function. As a special case of the NTS-*G* method, a NTS-Weibull distribution was considered. The estimation of the parameters of the NTS-*G* method was also discussed. Furthermore, a SS using the NTS-Weibull distribution was also carried out. A practical application of the NTS-Weibull distribution from the healthcare sector was considered. The practical illustration showed that the NTS-Weibull distribution was a suitable model for analyzing the medical data set. Furthermore, a new control chart based on the NTS-Weibull distribution was also discussed. The development of the control chart of the NTS-Weibull distribution was the first work ever done using the probability distributions that are introduced based on any trigonometric function.

Future work includes the development of the acceptance sampling for the NTS-Weibull distribution, bivariate and multivariate extensions, Bayesian estimation, and censored data analysis.

## Supporting information

S1 Appendix (PDF)
