# Supplementary material for: A new statistical methodology using the sine function: Control chart with an application to survival times data
Source: PLoS One. 2023 Aug 17;18(8):e0285914. doi: 10.1371/journal.pone.0285914 (PMC10434955; doi:10.1371/journal.pone.0285914)
Supplement: S1 Appendix — (PDF) [file pone.0285914.s001.pdf]

Appendix A

The following R-code is used to carry out the analysis in Section 5.

```
data=c(0.08, 2.09, 3.48, 4.87, 6.94, 8.66, 13.11, 23.63, 0.20, 2.23,
3.52, 4.98, 6.97, 9.02, 13.29, 0.40, 2.26, 3.57, 5.06, 7.09, 9.22,
13.80, 25.74, 0.50, 2.46, 3.64, 5.09, 7.26, 9.47, 14.24, 25.82, 0.51,
2.54, 3.70, 5.17, 7.28, 9.74, 14.76, 26.31, 0.81, 2.62, 3.82, 5.32,
7.32, 10.06, 14.77, 32.15, 2.64, 3.88, 5.32, 7.39, 10.34, 14.83,
34.26, 0.90, 2.69, 4.18, 5.34, 7.59, 10.66, 15.96, 36.66,  1.05, 2.69,
4.23, 5.41, 7.62, 10.75, 16.62, 43.01, 1.19, 2.75, 4.26, 5.41, 7.63,
17.12, 46.12, 1.26, 2.83, 4.33, 5.49, 7.66, 11.25, 17.14, 79.05,
1.35, 2.87, 5.62, 7.87, 11.64, 17.36, 1.40, 3.02, 4.34, 5.71, 7.93,
11.79, 18.10, 1.46, 4.40, 5.85, 8.26, 11.98, 19.13, 1.76, 3.25,  4.50,
6.25, 8.37, 12.02, 2.02, 3.31, 4.51, 6.54, 8.53,12.03, 20.28, 2.02,
3.36, 6.76, 12.07, 21.73, 2.07, 3.36, 6.93, 8.65, 12.63, 22.69)
###########################################################################
##### PDF
###########################################################################
pdf_pm <- function(par,x)
{
del= par[1]
alp= par[2]
lam= par[3]

((pi*lam*(lam-1)*alp*del*(x^(del-1))*exp(-alp*x^(del))*
cos((pi/2)*(1-exp(-alp*x^(del)))))/
(2*((lam-sin((pi/2)*(1-exp(-alp*x^(del)))))^2)))

}
###########################################################################
##### CDF
###########################################################################
cdf_pm <- function(par,x)
{
del= par[1]
alp= par[2]
lam= par[3]

1-((lam*(1-sin((pi/2)*(1-exp(-alp*x^(del))))))/
(lam-sin((pi/2)*(1-exp(-alp*x^(del))))))

}
set.seed(0)
goodness.fit(pdf=pdf_pm, cdf=cdf_pm,
starts = c(1,1,1.1), data = data,
method="SANN", domain=c(0,Inf),mle=NULL)
```
